# *De Novo* Assembly and Comparative Transcriptome Analyses of Red and Green Morphs of Sweet Basil Grown in Full Sunlight

**DOI:** 10.1371/journal.pone.0160370

**Published:** 2016-08-02

**Authors:** Sara Torre, Massimiliano Tattini, Cecilia Brunetti, Lucia Guidi, Antonella Gori, Cristina Marzano, Marco Landi, Federico Sebastiani

**Affiliations:** 1 Institute for Sustainable Plant Protection, Department of Biology, Agriculture and Food Sciences, The National Research Council of Italy, Sesto Fiorentino, Italy; 2 Trees and Timber Institute, Department of Biology, Agriculture and Food Sciences, The National Research Council of Italy, Sesto Fiorentino, Italy; 3 Department of Agri-Food Production and Environmental Sciences, University of Florence, Sesto Fiorentino, Italy; 4 Department of Agriculture, Food and Environment, University of Pisa, Pisa, Italy; Oregon State University, UNITED STATES

## Abstract

Sweet basil (*Ocimum basilicum*), one of the most popular cultivated herbs worldwide, displays a number of varieties differing in several characteristics, such as the color of the leaves. The development of a reference transcriptome for sweet basil, and the analysis of differentially expressed genes in acyanic and cyanic cultivars exposed to natural sunlight irradiance, has interest from horticultural and biological point of views. There is still great uncertainty about the significance of anthocyanins in photoprotection, and how green and red morphs may perform when exposed to photo-inhibitory light, a condition plants face on daily and seasonal basis. We sequenced the leaf transcriptome of the green-leaved Tigullio (TIG) and the purple-leaved Red Rubin (RR) exposed to full sunlight over a four-week experimental period. We assembled and annotated 111,007 transcripts. A total of 5,468 and 5,969 potential SSRs were identified in TIG and RR, respectively, out of which 66 were polymorphic *in silico*. Comparative analysis of the two transcriptomes showed 2,372 differentially expressed genes (DEGs) clustered in 222 enriched Gene ontology terms. Green and red basil mostly differed for transcripts abundance of genes involved in secondary metabolism. While the biosynthesis of waxes was up-regulated in red basil, the biosynthesis of flavonols and carotenoids was up-regulated in green basil. Data from our study provides a comprehensive transcriptome survey, gene sequence resources and microsatellites that can be used for further investigations in sweet basil. The analysis of DEGs and their functional classification also offers new insights on the functional role of anthocyanins in photoprotection.

## Introduction

The green color is most frequently associated to land plants, as early land plants evolved from green Chlorophytes [1]. However, the radiation of Angiosperms, dated approximately 200 MYA, was accompanied by a variety of colors that characterize actual plant landscapes. In contrast to the chromatic variations that characterize plant reproductive organs, leaves are usually green and this is not surprising given the optical properties of chlorophyll. Nonetheless, red leaves or red leaf portions are observed frequently in a range of species, particularly at specific developmental stages (e.g., in juvenile and senescence stages, [2,3]) or in response to different abiotic stresses [4–6]. Red coloration may be also a constitutive trait in some species, likely as the result of selection for aesthetic purposes [7].

Anthocyanins are the pigments responsible for leaf red coloration in the majority of plants, although in some members of Caryophyllales, betalains [8] confer red coloration to leaves. Anthocyanins have the potential to serve multiple functional roles in plants challenged against a wide range of stress agents of abiotic and biotic origin [3,9–12]. Epidermal anthocyanins confer greater capacity to red than to green individuals to withstand a severe, even transient excess of solar irradiance, because of their ability to absorb over the blue and the green portions of the solar spectrum [13–15]. Red varieties have received increasing interest over the last decade because of substantially greater content of health promoting substances as compared to green varieties, especially when plants grow under limited light irradiance. Genes encoding enzymes as well as regulatory genes involved in flavonoid and anthocyanin biosynthesis, have been characterized [16,17]. This has not resulted, however, into a corresponding in-depth knowledge about how cyanic and acyanic individuals of the same species perform when suffering from supernumerary photons, a condition plant face on daily, not only on seasonal basis [18].

Genomic and transcriptomic tools may help unveil the transcriptional regulation of genes involved in light-induced metabolic and signaling pathways, in leaves that differ in their ability to absorb over the visible portion of the solar spectrum [19]. Rapid advances in next-generation sequencing (NGS) technologies and associated bioinformatics tools provide novel opportunities for gene expression analysis [20]. The high-throughput RNA-sequencing (RNA-Seq) has emerged as a powerful method for transcriptome analysis of a complete set of expressed mRNA sequences in specific tissues as well as in the whole organism, and may have important applications in plant biology [21–23]. Since RNA-Seq is independent on prior knowledge of gene sequences, the reconstruction of transcriptome has key significance for molecular and genetic studies in non-model species [24,25]. *De novo* transcriptome analysis may help dissect mechanisms of photoprotection in acyanic and cyanic individuals, in the absence of mutants defective in early steps of the anthocyanin branch-pathway.

Our study explores the transcriptome of sweet basil (*O*. *basilicum*) one of the most popular cultivated herbs worldwide that displays a huge number of varieties differing in the leaf shape and aroma, as well as in the color of the leaf [26]. Recently, NGS technologies have been applied in sweet basil [27], but RNA-Seq analysis of cultivars that differs in the leaf color is still lacking. In particular, we applied RNA-Seq technology for in-depth sequencing of purple-leaved “Red Rubin” and green-leaved “Tigullio” transcriptomes, to provide adequate genomic resources for further investigation of the functional role of anthocyanins in photoprotection. We obtained about 128 million clean pair-end reads, which were further used for the *de novo* assembly of transcriptome of *O*. *basilicum*. The sequencing coverage was comprehensive enough to identify most unigenes and major metabolic pathways.

## Results and Discussion

### Sample preparation and Illumina sequencing

In our study, we carried out the RNA-Seq of four cDNA libraries, constructed from leaves of Red Rubin (RR) and Tigullio (TIG) basil cultivars, which were further sequenced with the Illumina platform. We obtained 128,551,512 paired-end (PE) clean reads by filtering and removing adapter sequences from raw data (Table 1). The output was greater than a previous study on sweet basil transcriptome which generated a total of about 50 millions high quality reads [27]. All clean reads derived from our analysis have been deposited at the Sequence Read Archive (SRA) of the National Centre of Biotechnology Information (NCBI) under the accession number SRA313233.

**Table 1:** Assembly statistics for Trinity RR-TIG.

	Trinity
**Reads**	128,551,512
**N50**	1,613
**N75**	2,488
**GC content**	41.3%
**Shortest transcript length**	201
**Longest transcript length**	15,660
**Mean Size**	988
**Total assembled bases**	166,663,118
**Count**	168,627
**Count (after CD-HIT 95%)**	134,194
**Count (after IsoPct selection)**	111,007

### *De novo* assembly of the transcriptome and assessment of assembly

Clean reads of RR and TIG were assembled *de novo* to generate a non-redundant dataset of gene sequences for sweet basil. We used individual reads of RR and TIG to produce *de novo* transcriptome assembly of each cultivar (S1 Table).

The assembly of *de novo* transcriptomes was performed with the de Bruijn's graph approach, which is particularly suitable for transcript reconstruction using Illumina reads [28]. Full-length transcripts reconstruction was carried out with Trinity, CLC Genomics Workbench (CLC), and SOAPdenovo-trans assemblers (all these assemblers adopt de Bruijn's graph approach). Trinity has been widely used for full-length transcripts reconstruction, but the use of a single k-mer (size = 25) may both introduce chimeric assemblies and be unable to cover the whole breadth of transcriptome expression [29]. CLC and SOAPdenovo-trans assemblers use k-mer of different sizes, thus providing a wider representation of different transcript isoforms and reducing perturbation graph topology that is originated from noises in the library sequencing [30]. In our study, k-mer sizes of 45-mer, 41-mer and 37-mer were best fitted for de Bruijn graph construction of for RR, TIG and RR-TIG, respectively.

In our study, CLC assembled 128,684 contigs, with a mean length of 658 bp and N50 of 962 bp (S1 File); SOAPdenovo-trans assembled 294,479 total transcripts, with a mean length of 352bp and N50 of 607 bp (S2 File), and Trinity yielded 168,627 transcripts, with a mean size of 988 bp and N50 of 1,613 bp. The quality of individual *de novo* assemblers was assessed based upon the length-weighted medians (N50 and N75), the number of both returned contigs and conserved core eukaryotic proteins (CEGMA, complete and partial proteins, [31]). We identified more than 87% highly conserved CEGMA core eukaryotic genes in each transcriptome, which supports assembly completeness. The number of conserved CEGMA proteins was higher in Trinity and, as expected, similar results were observed for the *de novo* assembly of the transcriptomes in each basil cultivar (S1 Table). Trinity turned out the most suitable *de novo* assembly program for our datasets, with higher N50 and N75 statistics and transcript numbers, and recovering more full-length transcripts of higher quality. Therefore, Trinity transcripts were retained for further analyses, confirming previous reports by Villarino et al. [32] and Grabherr et. al. [33]. We observed that a large percentage of transcripts were small-sized, since 36.4% of contigs were in the 200–400 bp size range (S1 Fig). Transcripts were therefore clustered using a sequence similarity threshold of 95%, to join further sequences and remove any redundant and/or highly similar contigs. The resulting 134,194 transcripts were further processed based on reads abundance, by removing contigs with < 1% of reads per component (Table 1, S3 File). The final unigene dataset consisted of 111,007 transcripts.

### Annotation and characterization of *de novo* transcripts

Transcripts were subjected to BLASTX homology search against the RefSeq plant database at the National Center for Biotechnology Information (NCBI, http://www.ncbi.nlm.nih.gov/) and the *Arabidopsis* protein database, allowing annotation of 91,231 (82.2%) and 57,663 (51.9%) transcripts, respectively (S2 Table). The percentage of annotated unigenes was similar to previous study in *O*. *basilicum* (81.1%) [27], suggesting that the assembled unigenes have captured the majority of sweet basil transcriptome. The E-value distribution of the top hits from RefSeq plant database indicates that 57.5% has strong homology (E-value smaller than 1.0e⁻⁵⁰), while 41.6% of homologous hits has E-value ranging from 1.0e⁻⁵ to 1.0e⁻⁵⁰ (S2 Fig). Similarity distribution (S2 Fig) shows that 44.6% of the query sequences had similarity > 80%, whereas similarity ranged from 40% to 80% in 55.3% of hits. About 52% of annotated transcripts of purple and green basil were assigned (with the best score), to the sequences of top four species, namely *Sesamum indicum* (40.7%), *Nicotiana sylvestris* (4.2%), *Nicotiana tomentosiformis* (4.0%) and *Vitis vinifera* (2.9%) (S2 Fig). Approximately, 28% of annotation descriptions against RefSeq were uninformative, containing “hypothetical” (7234) or “uncharacterized” (18,601) terms, likely due to insufficient knowledge of *O*. *basilicum* genome. The high rate (49%) of sequences without a significant homologous hit and informative description (as compared with other non-model plant species, [34–36]) can be partly due to both potentially novel sequences not yet reported in other crop species and the occurrence of highly divergent genes.

**Fig 1:**
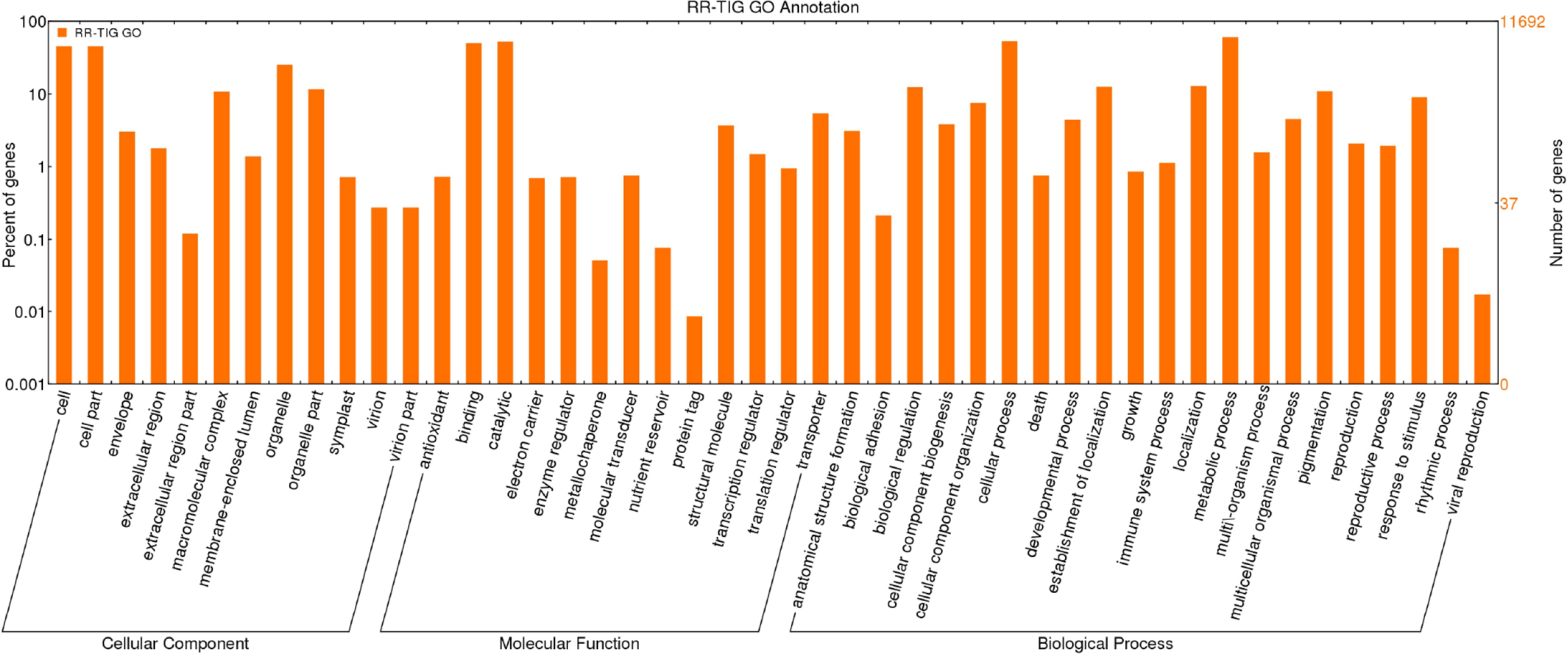
Gene Ontology assignments for RR-TIG transcriptome. The results are summarized in terms of three functional categories: cellular component, molecular function and biological process. The GO terms were visualized using WEGO (http://wego.genomics.org.cn).

BLASTX hits were used for transcript mapping and subsequent assignment of gene ontology (GO) annotations, using Blast2GO PRO. Among the 29,941 transcripts with at least one mapped GO term, only 11,692 were annotated and categorized into 46 functional groups, belonging to three main GO ontologies: molecular function (25.1%), cellular component (31.3%) and biological process (43.6%) (Fig 1, S4 File). This low rate of BLAST hits annotation to GO terms has been previously reported in sweet basil study of *de novo* transcriptome, where highest percentage of genes were classified under “unknown groups” [27], possibly because *O*. *basilicum* does not belong to model organisms family. “Catalytic activity” (6,151 transcripts; GO:0003824), “binding” (5,806; GO:0005488), and “transporter activity” (633; GO:0005215) are GO terms mostly represented in the molecular function category. Biological process are mostly enriched in “metabolic process” (7,028; GO:0008152), “cellular process” (6,231; GO:0009987), “localization” (1,504; GO:0051179) GO terms. Finally, GO terms. “cell” and “cell part” (5,295; GO:0005623, GO:0044464), “organelle” (2,938; GO:0043226), mostly represent the cellular component function category.

All transcripts were compared against InterPro database to find possible matches of domains/families. This annotation step allows retrieving protein domains that might be not accounted by simple sequence-based alignments. In Table 2 we show the ranking of 30 most abundant InterPro domains/families, based on the number of RR-TIG transcripts in each InterPro domain. About 42% of 6,411 domains/families had an occurrence of 1–3 sequences, whereas a small proportion gathered a high number of sequences. Among these protein domains/families, the most represented domain is IPR027417 (P-loop containing nucleoside triphosphate hydrolase) with 2,805 annotated transcripts, and "Protein kinase" and its subcategories containing “Serine/threonine-protein kinase", which are well-known regulators of cellular pathways. Moreover, highly represented are “Cytochrome P450” (Cyt_P450) and “WD40-repeat” domains, both associated with signal transduction and transcription, and “Zinc-finger related RING protein” (Znf-RING) domain.

**Table 2:** The 30 most representative GO terms revealed by the InterProScan annotation.

GO_ID	GO_Name	Aspect	Accession_Count
GO:0005524	ATP binding	Molecular_Function	4901
GO:0016772	transferase activity, transferring phosphorus-containing groups	Molecular_Function	2719
GO:0006468	protein phosphorylation	Biological_Process	2374
GO:0004672	protein kinase activity	Molecular_Function	2370
GO:0055114	oxidation-reduction process	Biological_Process	2328
GO:0016020	membrane	Cellular_Component	2259
GO:0003824	catalytic activity	Molecular_Function	2250
GO:0003676	nucleic acid binding	Molecular_Function	2204
GO:0016021	integral component of membrane	Cellular_Component	2046
GO:0008270	zinc ion binding	Molecular_Function	2006
GO:0003677	DNA binding	Molecular_Function	2003
GO:0008152	metabolic process	Biological_Process	1781
GO:0004674	protein serine/threonine kinase activity	Molecular_Function	1359
GO:0006355	regulation of transcription, DNA-templated	Biological_Process	1340
GO:0055085	transmembrane transport	Biological_Process	1270
GO:0005634	nucleus	Cellular_Component	1252
GO:0016491	oxidoreductase activity	Molecular_Function	1213
GO:0000166	nucleotide binding	Molecular_Function	1039
GO:0005622	intracellular	Cellular_Component	935
GO:0006508	proteolysis	Biological_Process	891
GO:0005975	carbohydrate metabolic process	Biological_Process	848
GO:0006810	transport	Biological_Process	788
GO:0046872	metal ion binding	Molecular_Function	758
GO:0003700	sequence-specific DNA binding transcription factor activity	Molecular_Function	718
GO:0005488	binding	Molecular_Function	670
GO:0005506	iron ion binding	Molecular_Function	666
GO:0020037	heme binding	Molecular_Function	643
GO:0003723	RNA binding	Molecular_Function	642
GO:0005737	cytoplasm	Cellular_Component	616
GO:0003735	structural constituent of ribosome	Molecular_Function	613

**Fig 2:**
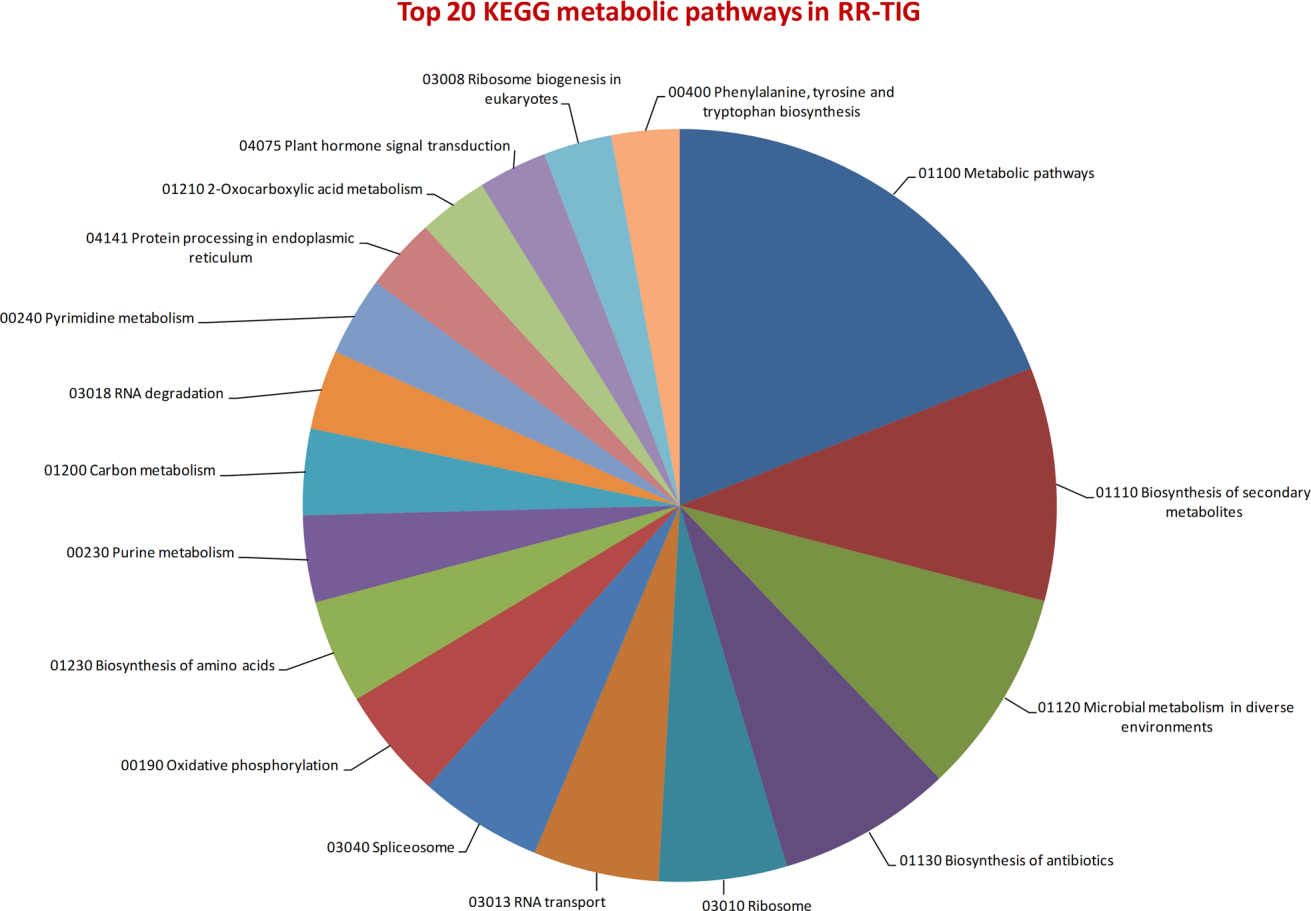
Top 20 identified KEGG pathways. The top 20 distribution of categorization of basil transcripts to KEGG biochemical pathways.

We conducted KEGG pathway/based analysis (on 111,007 RR-TIG transcripts) to investigate the biological functions of novel transcripts of sweet basil. Overall, 11,199 sequences were assigned to 170 KEGG pathways. The most represented pathways are “metabolic pathways” (8,1%), “biosynthesis of secondary metabolites” (4,2%), “microbial metabolism in diverse environment” (3.7%), “biosynthesis of antibiotics” (3.2%) and “ribosome” (2.3%) (Fig 2). The 633 transcripts in the “biosynthesis of secondary metabolites” category expressed in sweet basil cultivars may have interest in defining pathways of synthesis and turnover of compounds, which have potential beneficial effects to human health [37]. Thus, an accurate knowledge of basil secondary metabolism opens interesting opportunities for plant breeding. A total of 277 transcripts of flavonoid/anthocyanin metabolism were identified in the examined basil cultivars: glutathione metabolism (0.66%), phenylpropanoid biosynthesis (0.47%), phenylalanine metabolism (0.42%), flavonoid biosynthesis (0.15%), ABC transporters (0.11%), anthocyanin biosynthesis (0.03%) and flavone and flavonol biosynthesis (0.01%) (S3 Table). Data of our study provide, therefore, new insights to dissect molecular mechanisms that are at the base of flavonoid and anthocyanin accumulation in green and red basil morphs in response to high solar irradiance.

### Identification of SSR Markers

Simple sequence repeats (SSRs), also known as microsatellites, have been proved to be highly informative and widely used molecular markers for evolutionary and genetics studies. Within these well-established molecular markers, gene-derived SSRs are a valuable resource because they have higher rates of transferability between species as compared to random genomic SSRs. In this study, we predicted potential SSRs in the assembled unigenes of RR and TIG basil cultivars. By estimating the expression level of Trinity transcripts, unigenes of both cultivars with a low coverage (FPKM ≤ 1.5) were excluded to increase the reliability of SSRs identification. Thus, two sets of 45,244 and 51,493 unigenes for TIG and RR, respectively, were searched for repeat motifs to explore their SSRs profiles. A total of 5,468 and 5,969 potential SSRs have been identified in TIG and RR cultivars, respectively. Most SSR motifs are dinucleotides (3,089 and 3,381 in TIG and RR, respectively), accounting for 56.5% and 56.6% of all the predicted SSRs, followed by trinucleotides (2,257, 41.3% in TIG and 2,434, 40.8% in RR), tetranucleotides (95, 1.7% in TIG and 113, 1.9% in RR), and penta/hexanucleotides (5, 0.1% in TIG and 41, 0.7% in RR, S3 Fig). The most abundant repeat type is AG/CT, followed by AC/GT and AAG/CTT in both cultivars (S3 Fig). The overall frequency of SSRs (excluded mononucleotides) is around 1/12kb in both TIG and RR. Checking for potential polymorphisms between cultivar loci, microsatellites of two transcriptomes were compared through Blast search: 406 unigenes have been identified as the same loci, having the same flanking sequences and the same type of repeat motifs. The alignments of the homologous SSRs-containing loci of RR and TIG identified *in silico* 66 polymorphic microsatellites (S4 Table). The cultivar specific identification of SSRs from RNA-Seq data provides a new way to develop polymorphic SSR markers and new potential tools for genetic diversity assessments, variety protection and molecular mapping.

### Comparative DEG profiling in RR and TIG grown in full sunlight

**Fig 3:**
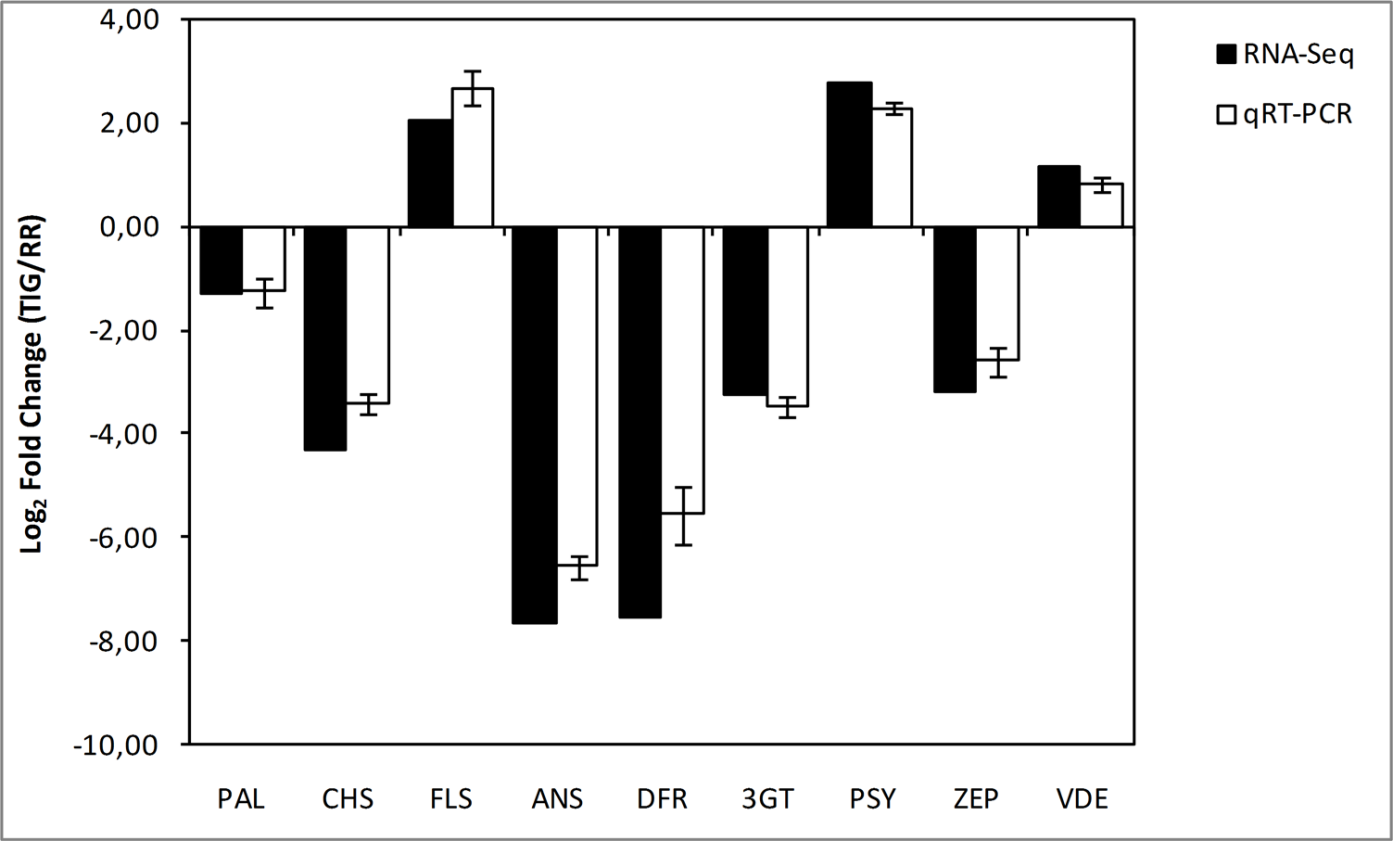
Validation of RNA-Seq data by qRT-PCR. Comparison of expression level of nine DEGs between RNA-Seq data and qRT-PCR assay. PCR primers and unigene IDs are listed in S7 Table.

The suitability of statistical analysis to identify differentially expressed genes (DEGs) was checked by a quality control test of overall reads distribution and variability (S4 Fig). Genes differentially expressed in the examined *O*. *basilicum* cultivars grown under full solar irradiance, were identified by aligning separately reads generated upon RR and TIG sequencing to the 111,007 transcripts, derived from RR-TIG total assembly. The abundance of gene expression was estimated as RPKM (reads per kilo base of exon model per million mapped reads, S5 File). A total of 2,372 transcripts has been identified as differentially expressed based on absolute fold change greater than 2 and P-value <0.05. There are 1,568 down-regulated and 804 up-regulated transcripts in green as compared to purple basil (S5 Table). To validate the RNA-Seq results, nine DEGs (PAL, CHS, FLS, ANS, DFR, 3GT, PSY, VDE, ZEP) with different expression patterns were selected for quantitative real-time PCR (qRT-PCR) analysis. All nine genes exhibited similar expression pattern to those obtained by sequencing (Fig 3). This offers further experimental validation to the reliability of our RNA-Seq analysis.

Among the BLAST hits of the 2,372 DGEs, approximately 28% are uninformative (“not available”, “predicted protein”, “uncharacterized” and “hypothetical”). The potential functions of DEGs were further examined using the Gene Ontology classification system. This annotation reveals 29 functional groups, which are distributed under the following categories: 14 Biological Process, 5 Cellular Component and 10 Molecular Function (GO level 4, S5 Fig). Nine GO categories are only down-regulated in TIG: e.g. “ribonucleprotein complex”, “anthocyanin metabolic process” and “positive regulation of biosynthetic process”. There are four GO-categories that are uniquely up-regulated in green basil, and corresponds to “glucosinolate metabolic process”, “chlorophyll metabolic process”, “terpenoid metabolic process” and “enzyme regulator”. GO categories such as “phenylpropanoid metabolic process”, “pigment biosynthetic process”, “secondary metabolic process” have been identified in both up- and down-regulated expressed genes.

**Fig 4:**
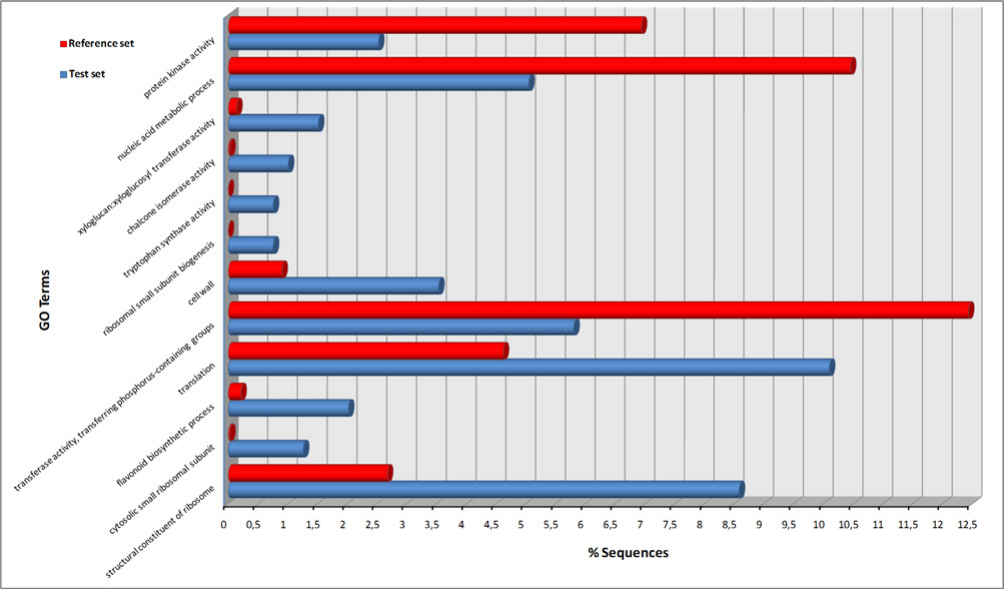
Enriched GO terms for RR-TIG DGEs. The bar chart shows for each significant GO termthe amount (percentage) of annotated transcripts Blue bars correspond to the sequences of the test-set and red bars correspond to the reference transcriptome dataset.

Genes differentially expressed between the red and green morphs of sweet basil were subjected to functional gene enrichment analysis (GEA) to explore whether specific categories are over- or under- represented compared to the reference transcriptome. We identified 222 GO functional categories that were significantly enriched in DEGs (p-value <0.05, see for full list of GO terms S6 Table). After using the blast2GO tool to reduce the GO enriched terms, the most specific terms (the highest level GO terms of a parental line) are reported in Fig 4. Among the significantly enriched GO terms, DEGs involved in “flavonoid biosynthetic process” (GO:0009813) and “chalcone isomerase activity” (GO:0045430) are noteworthy and suggest a possible relevant role of these secondary metabolites in the acclimation strategies adopted by the two basil morphs to high solar irradiance.

**Fig 5:**
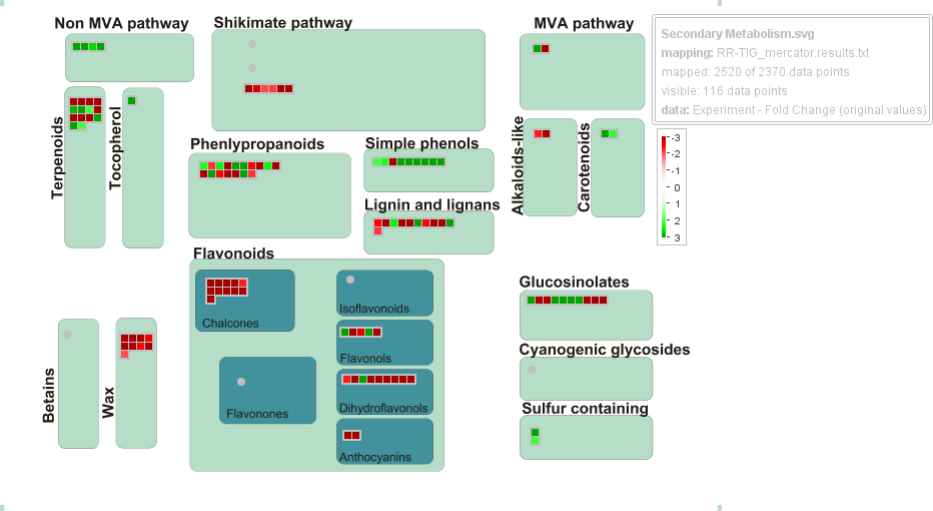
MapMan-based visualization of the DEGs involved in “secondary metabolism”. In the display, each BIN or subBIN is represented as a block where each transcript is displayed as a square, which is either colored red if this transcript is up-regulated in RR or green if this transcript is up-regulated in TIG.

A detailed functional analysis of genes differentially expressed in the examined basil cultivars, which was performed with the MapMan software, allowed drawing an overview of DEGs involved in various metabolic pathways (Fig 5). Some of the DEGs discussed in this section are summarized in Table 3. The MapMan diagram of secondary metabolism in sweet basil shows that all transcripts coding for enzymes of the MEP pathway, referred as to non-mevalonate pathways (Fig 5), are significantly up regulated in TIG. These mostly regard enzymes (or genes) involved in the early committed steps of the MEP pathway, namely 1-deoxy-D-xylulose-5-phosphate (DOXP) synthase (DXS) and DOXP reducto-isomerase (DXR) (Table 3). In contrast, all transcripts of the shikimate pathway are up-regulated in RR.

**Table 3:** DEGs identified by comparing RR and TIG basil varieties.

Transcript ID	Length (bp)	Description (Blast hit)	RR—RPKM	TIG—RPKM	Fold Change
***MEP pathway***					
comp43024_c0_seq1	2379	1-deoxy-D-xylulose-5-phosphate synthase (DXS)	6.25	31.47	5.03
comp49578_c0_seq12	3470	1-deoxy-D-xylulose 5-phosphate reductoisomerase (DXR)	5.55	19.50	3.51
***Carotenoids***					
comp48796_c1_seq3	1345	phytoene synthase (PSY)	6.52	44.31	6.80
comp50273_c0_seq2	1927	violaxanthin de-epoxidase (VDE)	20.10	44.72	2.23
***Tocopherol***					
comp48509_c0_seq4	1254	4-hydroxyphenylpyruvate dioxygenase (HDP)	4.31	13.30	3.09
***Waxes***					
comp35858_c1_seq1	827	protein ECERIFERUM 1-like (CER1)	9.49	0.52	-18.23
comp39531_c1_seq1	1761	protein ECERIFERUM 1-like (CER1)	28.35	11.49	-2.47
comp45710_c0_seq1	2116	protein ECERIFERUM 1-like (CER1)	206.70	68.03	-3.04
comp47826_c2_seq1	2001	protein ECERIFERUM 1-like (CER1)	15.28	1.18	-12.92
comp45316_c1_seq9	1011	protein ECERIFERUM 3-like (CER3)	9.17	0.64	-14.37
comp47720_c1_seq2	1357	3-ketoacyl-CoA synthase 6-like (KCS6)	57.90	18.63	-3.11
comp45887_c5_seq2	2127	O-acyltransferase WSD1-like (WSD1)	13.80	3.49	-3.95
***Flavonoids***					
comp39915_c0_seq2	1722	flavonol synthase (FLS)	4.32	18.31	4.24
comp30546_c3_seq1	1434	flavonol synthase (FLS)	66.87	150.05	2.24
comp40361_c0_seq2	1380	flavonol synthase (FLS)	49.18	108.52	2.21

Members of Lamiaceae, especially basil, display leaf surfaces rich in peltate glands (trichomes) that synthesize phenyl-propenes, mono- and sesqui-terpenes that impart distinct flavors and aromas [38]. The MEP and MVA pathways produce isopentenyl diphosphate and its isomer dimethyl-allyl diphosphate, which are precursors of terpene biosynthesis [39]. The deviation of upstream metabolites into the MEP pathway and the reduced metabolic flow into the shikimate/phenylpropanoid pathway observed in green basil, possibly as the result of breeding practices designed to select plant rich in aromatic compounds, may have great impact on the plant’s ability to cope with pathogen and predator attacks [40].

As expected, most genes involved in the biosynthesis of anthocyanins are up regulated in RR, the reverse being the case of genes involved in carotenoid (phytoene synthase (PSY), violaxanthin de-epoxidase (VDE) and tocopherol biosynthesis (4-hydroxyphenylpyruvate dioxygenase (HDP)) (Table 3). Carotenoids, particularly xanthophylls play key roles in photoprotection, by dissipating excess radiant energy through non-photochemical quenching as well as behaving as chloroplast antioxidants [41]. This observation taken together with the up-regulation of tocopherol (a well-known chloroplast antioxidant, [42]) biosynthesis, suggests that green basil likely suffered from photo-oxidative stress to greater degree than purple basil did, as previously reported for red and green morphs of several species (for recent review articles, see [2,12]). Data of our study, therefore, add further experimental validation to previous suggestions that anthocyanins may play an effective role in photoprotection [12]. It is also worth noting that the entire cluster of genes related to the synthesis of waxes (Fig 5), CER1 (ECERIFERUM 1, [43]), CER3 [44], KCS6 [45] and WSD1 [46], is greatly over-expressed in RR (Table 3). Waxes are involved in avoiding the entry of photons in the leaf by enhancing reflectance of incident light by leaf surfaces [47]. In some species, epicuticular waxes may greatly contribute in reflecting the shortest solar (UV) wavelengths (and to a lesser extent blue light, [48]), and this may have adaptive significance for plants growing under high solar irradiance [49].

**Fig 6:**
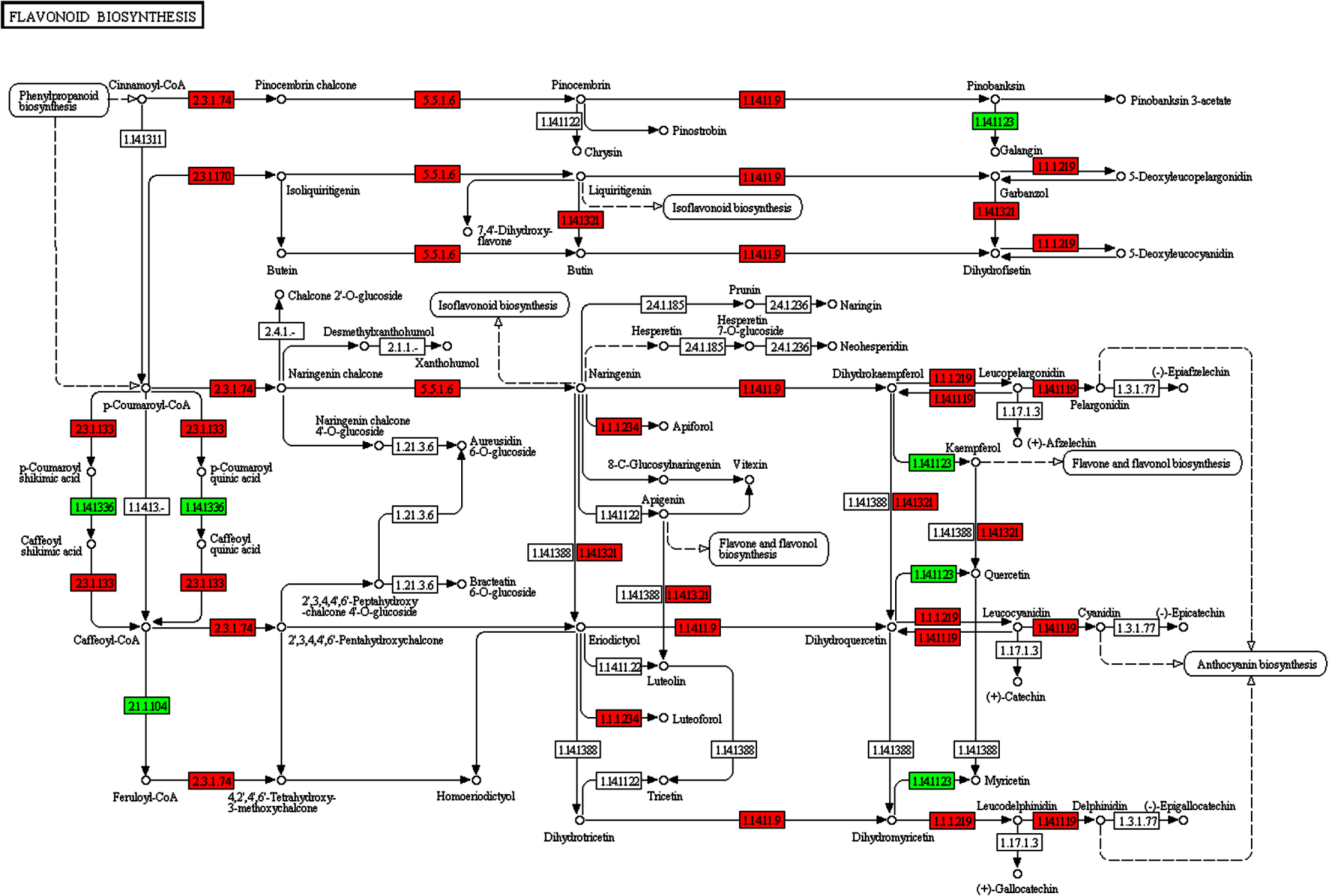
KEGG map of the DEGs involved in the flavonoid biosynthesis for the sweet basil leaf. Red boxes indicate overexpression in purple basil and green boxes overexpression in green basil.

We show that the flavonoid pathway, leading to the synthesis of flavones, flavonols and anthocyanins, greatly differs between purple and green-leaved basil [50], as revealed by DEGs mapping in KEGG pathways. In our annotated *O*. *basilicum* RR-TIG transcriptome dataset, 23 transcripts encoding most of the enzymes of flavonoid biosynthesis pathway were identified (S6 Fig). Nineteen of these enzymes (corresponding to nine ko numbers) are down-regulated in green as compared to purple basil (red boxes, Fig 6), consistent with the large flavonoid-anthocyanin production in purple basil. Nonetheless, three genes (corresponding to three ko numbers, green boxes) are up-regulated in green basil. Interestingly, one of these genes corresponds to flavonol synthase (E.C. 1.14.11.23), which is known to catalyze the divergent conversion of dihydro-flavonols to produce flavonols instead of anthocyanins (Table 3). There is recent compelling evidence that the expression of flavonol synthase is inversely related with red coloration and dihydro flavonol reductase expression in crabapples leaves [51]. This is consistent with our MapMan analysis, which indeed shows that genes involved in the biosynthesis of effective UV-screening “simple phenols” and “flavonols” is over-expressed in TIG. Flavonoids, particularly flavonols, the biosynthesis of which is up-regulated because of high sunlight (in the presence or in the absence of UV radiation, [52,53]), play a role in avoiding and countering the photo-oxidative damage driven by an excess of UV radiation. This is of adaptive value in plants challenged against excess light stress on long-term basis. We therefore hypothesize that in RR, an increased biosynthesis of waxes may partially compensate for the reduced biosynthesis of flavonols, which display the greater capacity to absorb over the UV region of the solar spectrum.

## Conclusions

We investigate the transcriptome profile of red and green morphs of sweet basil using Illumina sequencing technology. A high quality transcriptome, consisting of 111,007 unigenes, was functional annotated to number of putative genes related to numerous metabolic and biochemical pathways. We detected a large number of SSR loci, which may constitute a valuable resource for further experimentation on genetic diversity, linkage mapping, and germplasm characterization analysis in *Ocimum* species (Lamiaceae). In our study, *de-novo* sequencing of purple-leaved “Red Rubin” and green-leaved “Tigullio” transcriptomes may give new insights on the response mechanisms of cyanic and acyanic leaves suffering from an excess of solar irradiance. A constitutively epidermal cyanic filter may effectively limit photo-oxidative stress by absorbing the excess of supernumerary photons as compared to acyanic leaves. Green morphs counter an excess of unabsorbed visible light by enhancing the thermal dissipation of radiant energy through a more active carotenoid, particularly xanthophyll biosynthesis. This photoprotective strategy coupled with and enhanced biosynthesis of tocopherols, equip chloroplast membranes in TIG with a more active network of antioxidant defenses.

## Materials and Methods

### Plant material, library preparation and sequencing

Seeds of *O*. *basilicum* cv. ‘Red Rubin’ (RR) and cv. ‘Tigullio’ (TIG) were purchased from Franchi Sementi (Milan, Italy) and voucher samples have been deposited at the Institute for Sustainable Plant Protection (CNR). Seedlings were grown in 1.25 L pots with a commercial potting mix over a 30-day-period, and grown for additional three weeks in screenhouses, constructed with roofs and walls, under full sunlight, in the presence or in the absence of UV radiation, following the experimental set-up reported in Agati et al. [54]. Three-week-old leaves of each cultivar were sampled from plants that grew under different light regimes at different hours of the day (09:00–12:00–15:00 hrs) and pooled together prior to analysis. This partly overcame the different potential of green and red morphs to absorb over the UV or visible portion of the solar spectrum. Samples were frozen in liquid nitrogen and stored at -80°C prior to RNA isolation. Total RNA was extracted from 100 mg of leaf tissue using RNeasy Mini Kit, Qiagen, (Valencia, CA, USA) following manufacturer protocol, with little modifications. Briefly, 10% (v/v) of 20% (w/v) N-lauroyl sarcosine was added to RLC buffer. The sample solution was incubated for 10 min at 70°C under vigorous shaking. Contaminating genomic DNA was removed by adding 2 units of DNase I (Ambion, Life Technologies, Gaithersburg, MD) to sample solution kept at 37°C for 30 min. The integrity of total RNA was determined by running samples on 1% agarose gel. The concentration and the quality of total RNA was evaluated using an Agilent 2100 Bioanalyzer (Agilent Technologies, Palo Alto, CA, USA). RNA with RNA Integrity Number (RIN) >7 was used for successive analyses. cDNAs were amplified according to the Illumina RNA-Seq protocol and sequenced using the Illumina HiSeq2000 system (Illumina, Inc., CA, USA). Transcriptome library for sequencing was constructed according to the Illumina TruSeq RNA library protocol outlined in “TruSeq RNA Sample Preparation Guide”. The Illumina reads were trimmed at the ends by quality scores on the basis of the presence of ambiguous nucleotides (typically N, maximum value = 2) using ERNE-FILTER (www.erne.sourceforge.net), a modified version of the PHRED/PHRAP Mott’s trimming algorithm (www.phrap.org/phredphrap/phred.html), using default parameters, with the exception of minimum-size errors-rate, which were fixed at 50 and 25, respectively. All sequences reads were then processed for quality assessment using the FastQC quality control tool (v0.10.0, [55]). In all libraries we maintained a phred-like quality score (Q-score) of 20 for downstream analysis. cDNA Illumina sequencing was performed at IGA Technology Services Srl Service Provider (Udine, Italy).

### *De novo* assembly

The transcriptome assembly was performed on RR (65,661,162) and TIG (62,890,350) paired reads. A total *de novo* assembly was also performed using combined RR and TIG reads (128,551,512), to increase transcript coverage and build-up a reference transcriptome of sweet basil. To select the optimal k-mer length for the assemblies, k-mer analysis was used as implemented in the code KmerGenie [56]. We used three *de novo* transcriptome assemblers: Trinity r2013-02-25, SOAPdenovo-Trans v1.01 and CLC Genomics Workbench v.8.5.1 (CLC-Bio, Aarhus, Denmark). The softwares have been developed for transcriptome assembly of short reads, using Brujin graph algorithm [57]. First, clean reads were split into 'k-mers' and then assembled to produce contigs, which were joined into scaffolds, further assembled through gap filling to reconstruct unigenes. The most suitable assembly was selected by comparing contig mean size, number of sequences (N50) and Core Eukaryotic Genes Mapping Approach (CEGMA) output [31] of tested assembly programs. We used contigs longer than 200 nt for further analyses. To reduce assembly redundancy the transcripts were clustered at 95% identity using CD-HIT-EST v4.6.1 [58]. Filtration of likely contig artifacts and low expressed contigs was conducted by preliminarily estimating reads abundance for each contig using RSEM (RNASeq by Expectation Maximization) software package [59], with Bowite2 [60] bam file output. Contigs representing more than 1% of the per-component (IsoPct) expression level were kept for further analyses.

### Annotation and characterization of the *de novo* transcripts

Analysis of sequence similarity was performed using BLAST (Basic Local Alignment Search Tool) algorithm with an E-value cut-off of 10^−5^ [61] and Reference Sequence (RefSeq) plant protein collection database to assess the similarity of Trinity clustering transcripts to those of other model and closely related species. We used Blast2GO suite [62] to generate gene ontology (GO) terms based on the RefSeq BLAST, and the WEGO software to visualize distribution of gene functions [63]. BLAST search against InterPro protein families database [64] retrieved putative functions of newly assembled transcripts. Finally, contigs resulting from Trinity assembly were submitted to the Kyoto Encyclopedia of Genes and Genomes (KEGG) Automatic Annotation Server (KAAS) (v1.6a) [65] to gain an overview of gene pathway networks. The alignment was performed using KAAS default settings with single-directional best hit (SBH) method and databases including all available plant organisms (*Arabidopsis thaliana*, *Arabidopsis lyrata*, *Theobroma cacao*, *Glycine max*, *Fragaria vesca*, *Cucumis sativus*, *Vitis vinifera*, *Solanum lycopersicum*, *Oryza sativa japonica*).

### Simple sequence repeats (SSRs) identification

SSRs mining and primers design were performed following the method described previously [66]. In this study, more than 5 times repeats of di-, tri-, tetra-, penta- and hexa-nucleotide were included in search criteria in MISA script (http://pgrc.ipk-gatersleben.de/misa/).

### Analysis of differentially expressed genes

CLC Genomics Workbench was used to map the reads to assemblies, and identify and analyze the differentially expressed genes (DEGs). Reads from each sample were aligned to RR-TIG transcriptome reference with a minimum and maximum insert size of 180 and 1000, respectively. The gene expression level was calculated using RPKM method (Reads per kilobase transcriptome per million mapped reads) [20]. A box plot and a density analysis were conducted using R software (version 3.2.3) to estimate whether the RPKM overall distributions were comparable. Differentially expressed genes were identified by comparing expression values between samples and using Kal’s Z-test of proportions [66] with corrected Bonferroni p values (fold change >|2| and p value <0.05). The Z-test calculates the difference in the proportion of reads observed between two conditions and compares whether each sample was drawn from the same distribution relying on an approximation of the binomial distribution by the normal distribution [67].

Gene enrichment analysis (GEA) was performed on DEGs by Blast2GO using Fisher’s exact test (p<0.05) in combination with False Discovery Rate (FDR) correction for multiple testing [68]. Most specific terms were obtained removing parent terms of already existing, statistically significant, child GO terms through filtering enriched GO terms with FDR adjusted p-value < 0.05.

Functional categorization and pathway visualization of DEGs was performed using both KEGG orthologs (using KAAS with default parameters, http://www.genome.jp/kegg/) and the MapMan tool [69]. A MapMan BIN file, with hierarchical ontology system for basil genes, was prepared using Mercator (default options, Blast_cutoff: 50 and IS_DNA; [70], by comparing transcripts against already-classified proteins.

### Validation of DEGs by qRT-PCR

Total RNA was extracted from red and green basil morphs as already reported described above and reverse-transcribed by using SuperScript® VILO cDNA Synthesis Kit (Invitrogen, Carlsbad, CA). Nine DEGs, were chosen for validation by quantitative real-time PCR (qRT-PCR) and specific primer pairs (S7 Table) were designed using Primer3 [71]. qRT-PCR was carried out in a StepOnePlus real-time PCR system (Applied Biosystems, Foster City, CA, USA) with SYBR green technologies (Power SYBR green PCR Master Mix; Applied Biosystems) according to the manufacturer’s instruction. Measurements were performed on three replicates and the products were verified by melting curve analysis. The relative expression level of the selected unigenes was calculated using the comparative ΔΔCt method [72], by normalizing the number of target transcripts to the reference *Tubulin* gene (S7 Table).

## Supporting Information

S1 Fig Transcript size distribution. Frequency histogram showing the distribution of transcript length in sweet basil.
(TIF)

S2 Fig Characteristics of BLAST search of RR-TIG transcripts against Reference Sequence (RefSeq) plant protein collection database. (A) The E-value distribution of BLAST hits for the assembled RR-TIG sequences with a cutoff of E-value < 10^−5^. (B) The similarity distribution of BLAST hit for the assembled RR-TIG sequences with a cutoff of E-value < 10^−5^. (C) The species distribution of the top BLAST hits for each transcript in the RR-TIG transcriptome assembly from Blast2GO.
(TIF)

S3 Fig The SSR mining results in sweet basil cultivars. A. The profiles of different SSR types in TIG (green) and RR (red). B. The distribution of repeat motifs in TIG (green) and RR (red).
(TIF)

S4 Fig Plot analysis of transcript expression values (RPKM) in basil cultivars for quality control. The RPKM overall distribution and variability of cDNA libraries/samples were similar, indicating that they were comparable for identification of differentially expressed genes (DEGs) at the transcriptome level. (A) A box plot analysis with original expression values; (B) A box plot analysis with normalized expression values; (C) Density plot showing the distribution of log_2_(RPKM) in RR and TIG.
(TIF)

S5 Fig Histogram presentation of GO classification of DEGs. The results are summarized in three main categories: cellular component, molecular function, and biological process.
(TIF)

S6 Fig RR-TIG transcripts annotated into the KEGG-flavonoid biosynthesis pathway. (TIF)

S1 File CLC assembled transcripts. Fasta file of basil transcripts *de novo* assembled with CLC Genomics Workbench v.8.5.1.
(FASTA)

S2 File SOAPdenovo-trans assembled transcripts. Fasta file of basil transcripts *de novo* assembled with SOAPdenovo-Trans v1.01.
(ZIP)

S3 File Trinity assembled transcripts. Fasta file of basil transcripts *de novo* assembled with Trinity r2013-02-25.
(ZIP)

S4 File GO terms annotation. List of GO terms associated to sweet basil transcripts.
(ZIP)

S5 File Expression data for all the transcripts in the comparison TIG vs RR. (ZIP)

S1 Table Summary of the transcriptome sequencing and assemblies. Statistics of k-mer processed assemblies with three methods (Clc, So, Tr).
(DOCX)

S2 Table Blastx annotation descriptions. Blastx annotation of all transcripts against plant RefSeq and *Arabidopsis* databases with E-values and transcripts sizes.
(XLSX)

S3 Table Summary of KEGG annotations of basil transcriptome. (DOCX)

S4 Table Polymorphic microsatellites identified by comparing RR and TIG SSRs. (XLSX)

S5 Table Differentially expressed genes between TIG and RR. (XLSX)

S6 Table Gene ontology functional enrichment analysis of the DEGs. (XLSX)

S7 Table Primer sequences used in the qRT-PCR assay. (XLSX)
