# Supplementary figures and images for: De Novo Assembly and Comparative Transcriptome Analyses of Red and Green Morphs of Sweet Basil Grown in Full Sunlight
Source: PLoS One. 2016 Aug 2;11(8):e0160370. doi: 10.1371/journal.pone.0160370 (PMC4970699; doi:10.1371/journal.pone.0160370)

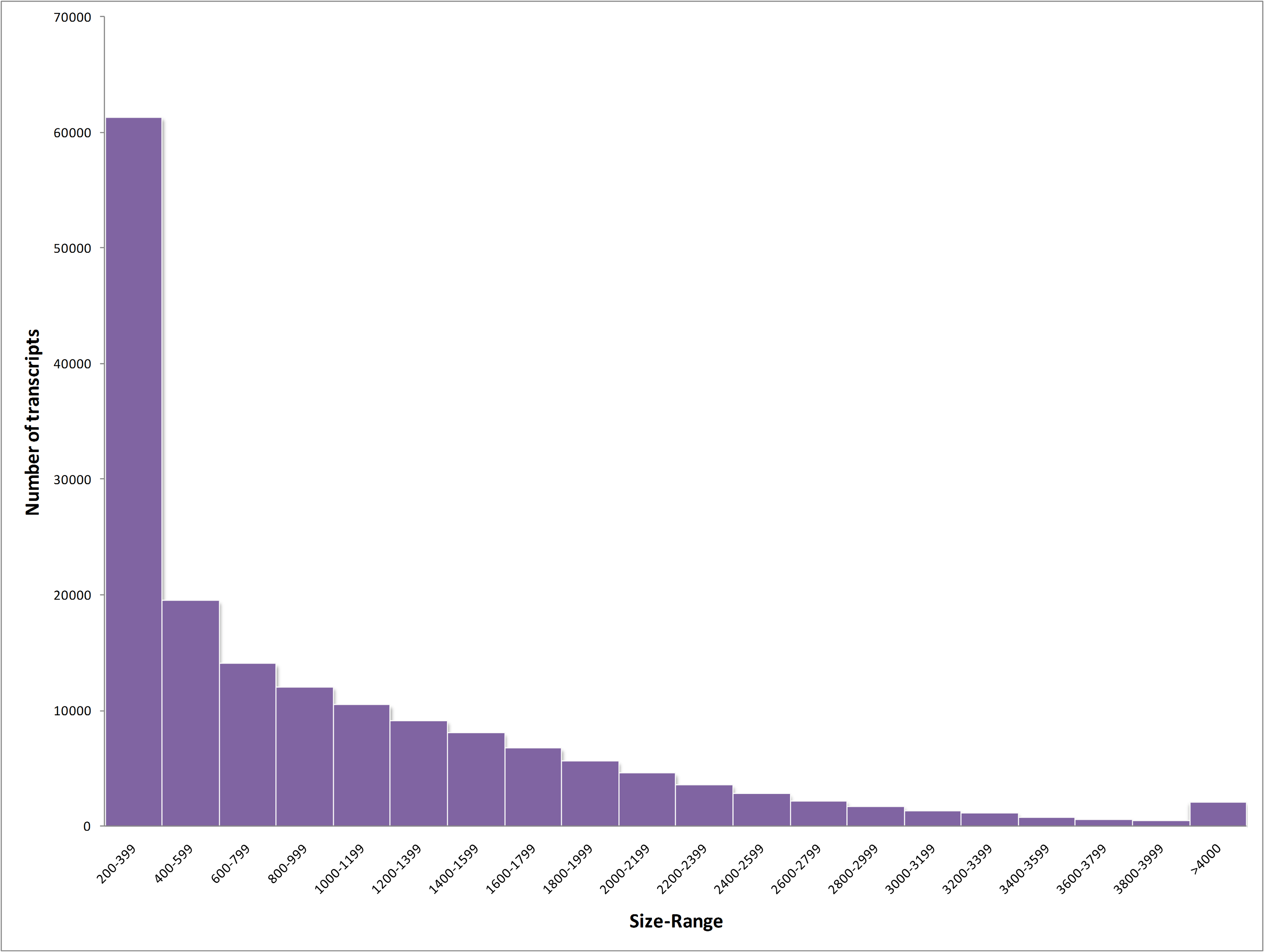

Supplement: S1 Fig — Frequency histogram showing the distribution of transcript length in sweet basil. (TIF) [file pone.0160370.s001.tif]

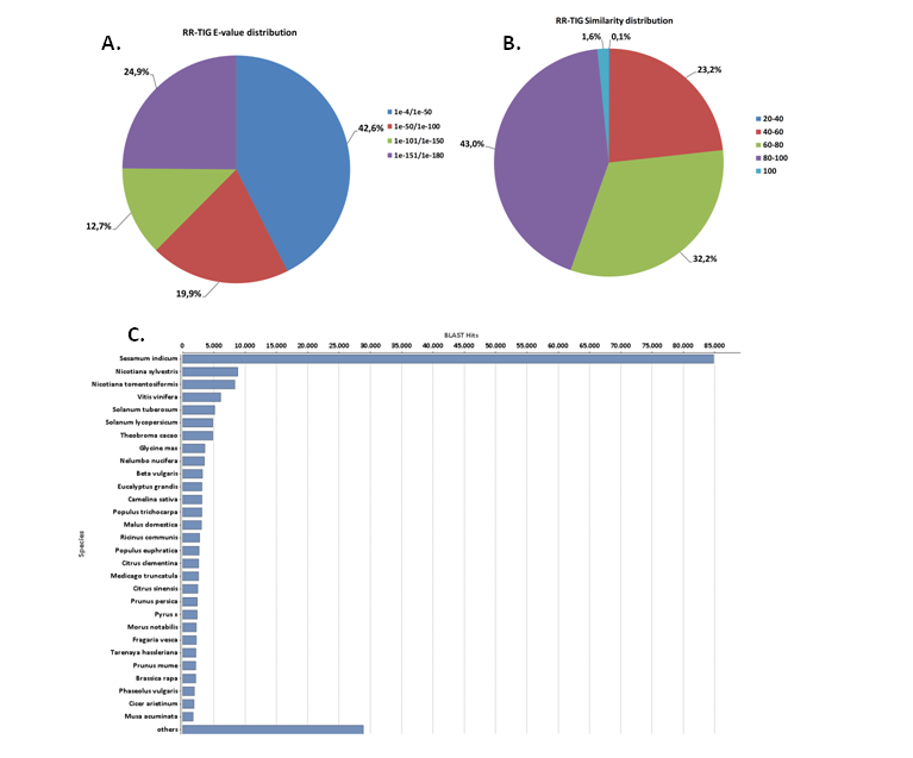

Supplement: S2 Fig — (A) The E-value distribution of BLAST hits for the assembled RR-TIG sequences with a cutoff of E-value < 10−5. (B) The similarity distribution of BLAST hit for the assembled RR-TIG sequences with a cutoff of E-value < 10−5. (C) The species distribution of the top BLAST hits for each transcript in the RR-TIG transcriptome assembly from Blast2GO. (TIF) [file pone.0160370.s002.tif]

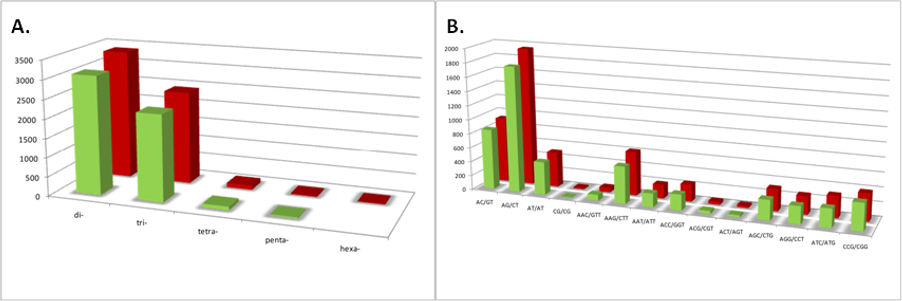

Supplement: S3 Fig — A. The profiles of different SSR types in TIG (green) and RR (red). B. The distribution of repeat motifs in TIG (green) and RR (red). (TIF) [file pone.0160370.s003.tif]

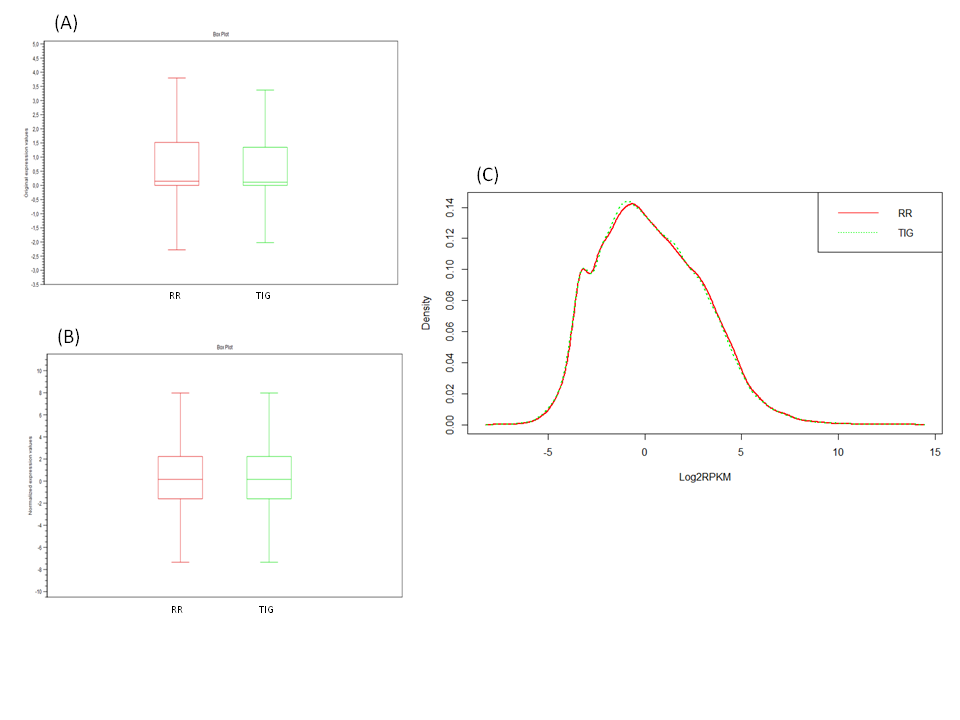

Supplement: S4 Fig — The RPKM overall distribution and variability of cDNA libraries/samples were similar, indicating that they were comparable for identification of differentially expressed genes (DEGs) at the transcriptome level. (A) A box plot analysis with original expression values; (B) A box plot analysis with normalized expression values; (C) Density plot showing the distribution of log2(RPKM) in RR and TIG. (TIF) [file pone.0160370.s004.tif]

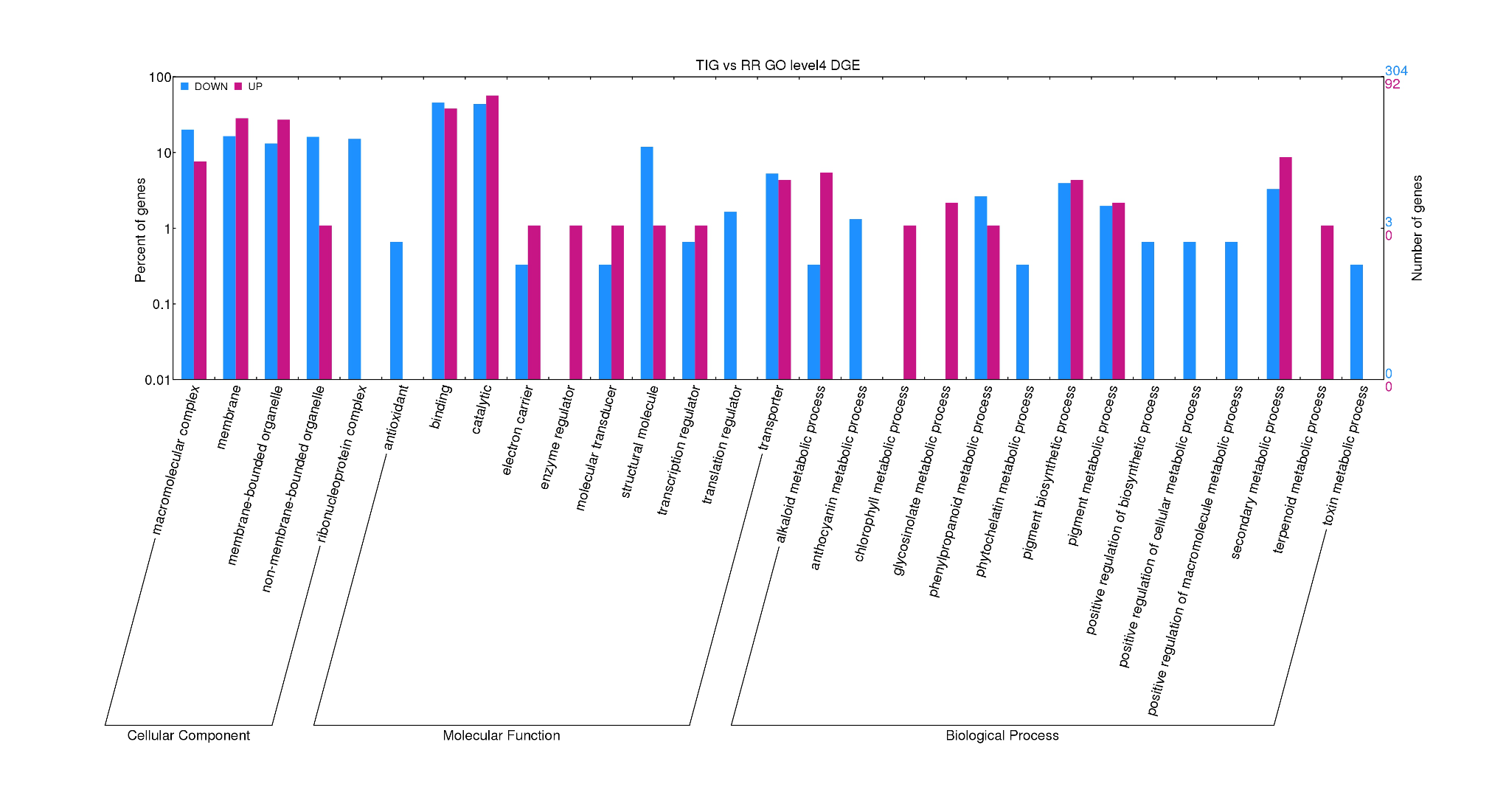

Supplement: S5 Fig — The results are summarized in three main categories: cellular component, molecular function, and biological process. (TIF) [file pone.0160370.s005.tif]

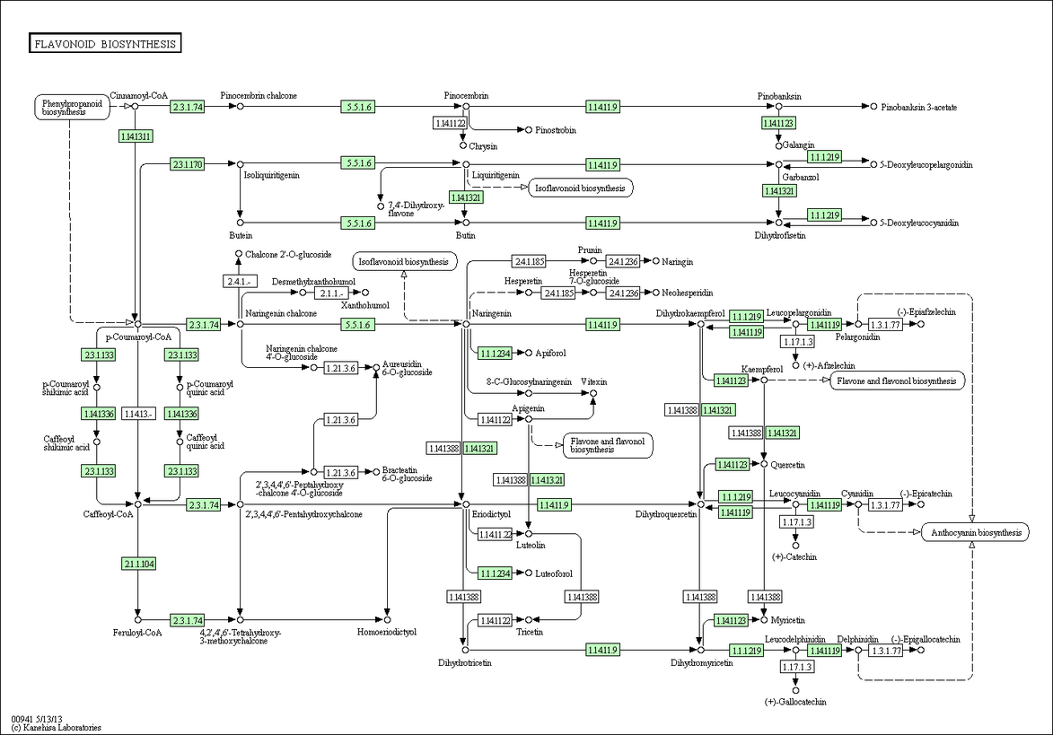

Supplement: S6 Fig — (TIF) [file pone.0160370.s006.tif]
