# Supplementary material for: De Novo Assembly and Comparative Transcriptome Analyses of Red and Green Morphs of Sweet Basil Grown in Full Sunlight
Source: PLoS One. 2016 Aug 2;11(8):e0160370. doi: 10.1371/journal.pone.0160370 (PMC4970699; doi:10.1371/journal.pone.0160370)
Supplement: S1 Table — Statistics of k-mer processed assemblies with three methods (Clc, So, Tr). (DOCX) [file pone.0160370.s012.docx]

| **S1 Table. Summary of the transcriptome sequencing and assemblies.** | | | | | | | | | | | |
| --- | --- | --- | --- | --- | --- | --- | --- | --- | --- | --- | --- |
| **RR** |  | **Reads** | **N50** | **N75** | **GC_Content** | **Shortest_Seq** | **Longest_Seq** | **Mean_Size** | **Count (including scaffolded regions)** | **% of complete CEGMA proteins** | **% of partial CEGMA proteins** |
|  | **CLCbio WB** | 65,661,162 | 1,098 | 1,846 | 42.76% | 98 | 13,490 | 729 | 95,336 | 80.24 | 97.18 |
|  | **SOAPdenovo-trans** |  | 1,727 | 2,692 | 42.98% | 100 | 15,743 | 740 | Total transcripts: 189,289; only scaffolds: 70,757 | 79.44 | 97.18 |
|  | **TRINITY** |  | 1,647 | 2,524 | 41.77% | 201 | 15,608 | 1,006 | Total transcripts: 132,147; Total components: 63,133 | 96 | 99.19 |
| **TIG** |  | **Reads** | **N50** | **N75** | **GC_Content** | **Shortest_Seq** | **Longest_Seq** | **Mean_Size** | **Count (including scaffolded regions)** | **% of complete CEGMA proteins** | **% of partial CEGMA proteins** |
|  | **CLCbio WB** | 62,890,350 | 1,127 | 1,943 | 42.40% | 93 | 15,560 | 746 | 81,179 | 68.95 | 91.53 |
|  | **SOAPdenovo-trans** |  | 1,570 | 2,535 | 42.01% | 100 | 15,738 | 537 | Total transcripts: 199,667; only scaffolds: 52,335 | 68.15 | 87.9 |
|  | **TRINITY** |  | 1,777 | 2,703 | 41.79% | 201 | 15,660 | 1,100 | Total transcripts: 125,603; Total components: 54,622 | 97.58 | 100 |
| **RR-TIG** |  | **Reads** | **N50** | **N75** | **GC_Content** | **Shortest_Seq** | **Longest_Seq** | **Mean_Size** | **Count (including scaffolded regions)** | **% of complete CEGMA proteins** | **% of partial CEGMA proteins** |
|  | **CLCbio WB** | 128,551,512 | 962 | 1,705 | 41.97% | 100 | 14,997 | 658 | 128,684 | 72.58 | 93.55 |
|  | **SOAPdenovo-trans** |  | 607 | 1,292 | 41.91% | 100 | 10,419 | 352 | Total transcripts: 294,479; only scaffolds: 56,891 | 61.69 | 91.53 |
|  | **TRINITY** |  | 1,613 | 2,488 | 41.31% | 201 | 15,660 | 988 | Total transcripts: 168,627; Total components: 77,889 | 97.58 | 99.6 |

Statistics of k-mer processed assemblies with three methods (Clc, So, Tr).
