# Supplementary material for: De Novo Assembly and Comparative Transcriptome Analyses of Red and Green Morphs of Sweet Basil Grown in Full Sunlight
Source: PLoS One. 2016 Aug 2;11(8):e0160370. doi: 10.1371/journal.pone.0160370 (PMC4970699; doi:10.1371/journal.pone.0160370)
Supplement: S3 Table — (DOCX) [file pone.0160370.s014.docx]

| **Additional file 2. The summary of KEGG annotations of basil transcriptome** | | | |
| --- | --- | --- | --- |
| **Pathway ID** | **Number of ko** | **Number of Genes** | **Percent** |
| 01100 Metabolic pathways | 381 | 1208 | 8.085 |
| 01110 Biosynthesis of secondary metabolites | 182 | 633 | 4.237 |
| 01120 Microbial metabolism in diverse environments | 129 | 559 | 3.741 |
| 01130 Biosynthesis of antibiotics | 134 | 475 | 3.179 |
| 03010 Ribosome | 103 | 348 | 2.329 |
| 03013 RNA transport | 92 | 342 | 2.289 |
| 03040 Spliceosome | 100 | 338 | 2.262 |
| 00190 Oxidative phosphorylation | 77 | 302 | 2.021 |
| 01230 Biosynthesis of amino acids | 98 | 282 | 1.887 |
| 00230 Purine metabolism | 80 | 236 | 1.580 |
| 01200 Carbon metabolism | 90 | 233 | 1.559 |
| 03018 RNA degradation | 49 | 216 | 1.446 |
| 00240 Pyrimidine metabolism | 68 | 215 | 1.439 |
| 04141 Protein processing in endoplasmic reticulum | 75 | 198 | 1.325 |
| 01210 2-Oxocarboxylic acid metabolism | 28 | 189 | 1.265 |
| 04075 Plant hormone signal transduction | 40 | 187 | 1.252 |
| 03008 Ribosome biogenesis in eukaryotes | 55 | 186 | 1.245 |
| 00400 Phenylalanine, tyrosine and tryptophan biosynthesis | 22 | 185 | 1.238 |
| 04144 Endocytosis | 39 | 184 | 1.232 |
| 04110 Cell cycle | 58 | 182 | 1.218 |
| 00561 Glycerolipid metabolism | 23 | 175 | 1.171 |
| 03030 DNA replication | 31 | 174 | 1.165 |
| 00510 N-Glycan biosynthesis | 30 | 171 | 1.145 |
| 03015 mRNA surveillance pathway | 44 | 170 | 1.138 |
| 00260 Glycine, serine and threonine metabolism | 32 | 154 | 1.031 |
| 00195 Photosynthesis | 38 | 153 | 1.024 |
| 03020 RNA polymerase | 29 | 149 | 0.997 |
| 03420 Nucleotide excision repair | 37 | 133 | 0.890 |
| 00350 Tyrosine metabolism | 18 | 133 | 0.890 |
| 03410 Base excision repair | 26 | 128 | 0.857 |
| 04120 Ubiquitin mediated proteolysis | 57 | 127 | 0.850 |
| 04142 Lysosome | 35 | 127 | 0.850 |
| 04114 Oocyte meiosis | 31 | 127 | 0.850 |
| 00563 Glycosylphosphatidylinositol GPI-anchor biosynthesis | 20 | 127 | 0.850 |
| 00600 Sphingolipid metabolism | 14 | 123 | 0.823 |
| 04146 Peroxisome | 36 | 114 | 0.763 |
| 00010 Glycolysis / Gluconeogenesis | 32 | 113 | 0.756 |
| 04626 Plant-pathogen interaction | 28 | 112 | 0.750 |
| 04330 Notch signaling pathway | 7 | 112 | 0.750 |
| 00450 Selenocompound metabolism | 8 | 111 | 0.743 |
| 00630 Glyoxylate and dicarboxylate metabolism | 28 | 104 | 0.696 |
| 00564 Glycerophospholipid metabolism | 34 | 102 | 0.683 |
| 04071 Sphingolipid signaling pathway | 18 | 102 | 0.683 |
| 00906 Carotenoid biosynthesis | 17 | 102 | 0.683 |
| 00100 Steroid biosynthesis | 16 | 102 | 0.683 |
| 00905 Brassinosteroid biosynthesis | 6 | 100 | 0.669 |
| 00330 Arginine and proline metabolism | 36 | 99 | 0.663 |
| 03050 Proteasome | 33 | 99 | 0.663 |
| 00480 Glutathione metabolism | 17 | 98 | 0.656 |
| 00520 Amino sugar and nucleotide sugar metabolism | 38 | 96 | 0.643 |
| 00592 alpha-Linolenic acid metabolism | 13 | 95 | 0.636 |
| 03440 Homologous recombination | 24 | 93 | 0.622 |
| 01212 Fatty acid metabolism | 29 | 91 | 0.609 |
| 04810 Regulation of actin cytoskeleton | 18 | 91 | 0.609 |
| 00051 Fructose and mannose metabolism | 19 | 90 | 0.602 |
| 04728 Dopaminergic synapse | 10 | 89 | 0.596 |
| 04152 AMPK signaling pathway | 26 | 88 | 0.589 |
| 04024 cAMP signaling pathway | 8 | 88 | 0.589 |
| 00750 Vitamin B6 metabolism | 5 | 88 | 0.589 |
| 03060 Protein export | 26 | 87 | 0.582 |
| 00290 Valine, leucine and isoleucine biosynthesis | 9 | 87 | 0.582 |
| 00900 Terpenoid backbone biosynthesis | 29 | 86 | 0.576 |
| 00500 Starch and sucrose metabolism | 30 | 82 | 0.549 |
| 00620 Pyruvate metabolism | 31 | 81 | 0.542 |
| 00030 Pentose phosphate pathway | 17 | 80 | 0.535 |
| 04922 Glucagon signaling pathway | 16 | 77 | 0.515 |
| 04130 SNARE interactions in vesicular transport | 19 | 74 | 0.495 |
| 00770 Pantothenate and CoA biosynthesis | 15 | 73 | 0.489 |
| 00710 Carbon fixation in photosynthetic organisms | 26 | 72 | 0.482 |
| 00130 Ubiquinone and other terpenoid-quinone biosynthesis | 20 | 71 | 0.475 |
| 00940 Phenylpropanoid biosynthesis | 17 | 71 | 0.475 |
| 04010 MAPK signaling pathway | 8 | 71 | 0.475 |
| 00908 Zeatin biosynthesis | 4 | 69 | 0.462 |
| 04014 Ras signaling pathway | 10 | 68 | 0.455 |
| 00513 Various types of N-glycan biosynthesis | 21 | 67 | 0.448 |
| 04210 Apoptosis | 6 | 67 | 0.448 |
| 00270 Cysteine and methionine metabolism | 32 | 66 | 0.442 |
| 00250 Alanine, aspartate and glutamate metabolism | 27 | 66 | 0.442 |
| 00970 Aminoacyl-tRNA biosynthesis | 25 | 63 | 0.422 |
| 00280 Valine, leucine and isoleucine degradation | 20 | 63 | 0.422 |
| 00020 Citrate cycle TCA cycle | 19 | 63 | 0.422 |
| 04510 Focal adhesion | 7 | 63 | 0.422 |
| 00360 Phenylalanine metabolism | 15 | 62 | 0.415 |
| 00860 Porphyrin and chlorophyll metabolism | 32 | 60 | 0.402 |
| 03022 Basal transcription factors | 30 | 60 | 0.402 |
| 00040 Pentose and glucuronate interconversions | 12 | 57 | 0.382 |
| 00061 Fatty acid biosynthesis | 16 | 56 | 0.375 |
| 04530 Tight junction | 10 | 56 | 0.375 |
| 04015 Rap1 signaling pathway | 5 | 55 | 0.368 |
| 00053 Ascorbate and aldarate metabolism | 16 | 53 | 0.355 |
| 00071 Fatty acid degradation | 12 | 53 | 0.355 |
| 04020 Calcium signaling pathway | 7 | 53 | 0.355 |
| 00562 Inositol phosphate metabolism | 21 | 50 | 0.335 |
| 00680 Methane metabolism | 19 | 48 | 0.321 |
| 00920 Sulfur metabolism | 14 | 48 | 0.321 |
| 03430 Mismatch repair | 22 | 47 | 0.315 |
| 00910 Nitrogen metabolism | 12 | 46 | 0.308 |
| 01040 Biosynthesis of unsaturated fatty acids | 12 | 46 | 0.308 |
| 00760 Nicotinate and nicotinamide metabolism | 12 | 43 | 0.288 |
| 00640 Propanoate metabolism | 14 | 40 | 0.268 |
| 04662 B cell receptor signaling pathway | 5 | 40 | 0.268 |
| 00340 Histidine metabolism | 11 | 39 | 0.261 |
| 00514 Other types of O-glycan biosynthesis | 3 | 39 | 0.261 |
| 04070 Phosphatidylinositol signaling system | 18 | 38 | 0.254 |
| 04712 Circadian rhythm - plant | 22 | 37 | 0.248 |
| 00062 Fatty acid elongation | 8 | 36 | 0.241 |
| 00410 beta-Alanine metabolism | 16 | 35 | 0.234 |
| 00740 Riboflavin metabolism | 6 | 34 | 0.228 |
| 04540 Gap junction | 4 | 34 | 0.228 |
| 00052 Galactose metabolism | 17 | 33 | 0.221 |
| 02020 Two-component system | 8 | 31 | 0.207 |
| 00310 Lysine degradation | 12 | 30 | 0.201 |
| 04621 NOD-like receptor signaling pathway | 4 | 30 | 0.201 |
| 04623 Cytosolic DNA-sensing pathway | 16 | 29 | 0.194 |
| 03320 PPAR signaling pathway | 10 | 29 | 0.194 |
| 00380 Tryptophan metabolism | 8 | 29 | 0.194 |
| 00565 Ether lipid metabolism | 7 | 26 | 0.174 |
| 00650 Butanoate metabolism | 10 | 25 | 0.167 |
| 00790 Folate biosynthesis | 10 | 25 | 0.167 |
| 00941 Flavonoid biosynthesis | 10 | 23 | 0.154 |
| 00300 Lysine biosynthesis | 9 | 23 | 0.154 |
| 00511 Other glycan degradation | 9 | 23 | 0.154 |
| 00780 Biotin metabolism | 8 | 22 | 0.147 |
| 04978 Mineral absorption | 6 | 21 | 0.141 |
| 00196 Photosynthesis - antenna proteins | 12 | 20 | 0.134 |
| 00531 Glycosaminoglycan degradation | 5 | 20 | 0.134 |
| 04122 Sulfur relay system | 9 | 18 | 0.120 |
| 00960 Tropane, piperidine and pyridine alkaloid biosynthesis | 8 | 18 | 0.120 |
| 00590 Arachidonic acid metabolism | 7 | 18 | 0.120 |
| 03450 Non-homologous end-joining | 8 | 16 | 0.107 |
| 02010 ABC transporters | 6 | 16 | 0.107 |
| 00073 Cutin, suberine and wax biosynthesis | 7 | 15 | 0.100 |
| 00904 Diterpenoid biosynthesis | 9 | 14 | 0.094 |
| 00903 Limonene and pinene degradation | 2 | 14 | 0.094 |
| 04974 Protein digestion and absorption | 3 | 13 | 0.087 |
| 00902 Monoterpenoid biosynthesis | 3 | 12 | 0.080 |
| 04744 Phototransduction | 2 | 12 | 0.080 |
| 04973 Carbohydrate digestion and absorption | 2 | 11 | 0.074 |
| 00540 Lipopolysaccharide biosynthesis | 8 | 10 | 0.067 |
| 00945 Stilbenoid, diarylheptanoid and gingerol biosynthesis | 5 | 10 | 0.067 |
| 00830 Retinol metabolism | 5 | 10 | 0.067 |
| 00909 Sesquiterpenoid and triterpenoid biosynthesis | 5 | 10 | 0.067 |
| 00603 Glycosphingolipid biosynthesis - globo series | 3 | 10 | 0.067 |
| 01220 Degradation of aromatic compounds | 3 | 9 | 0.060 |
| 00591 Linoleic acid metabolism | 4 | 6 | 0.040 |
| 00362 Benzoate degradation | 3 | 6 | 0.040 |
| 00627 Aminobenzoate degradation | 3 | 6 | 0.040 |
| 01062 Biosynthesis of terpenoids and steroids | 1 | 6 | 0.040 |
| 00942 Anthocyanin biosynthesis | 2 | 5 | 0.033 |
| 00966 Glucosinolate biosynthesis | 2 | 5 | 0.033 |
| 00364 Fluorobenzoate degradation | 1 | 5 | 0.033 |
| 00361 Chlorocyclohexane and chlorobenzene degradation | 1 | 5 | 0.033 |
| 00623 Toluene degradation | 1 | 5 | 0.033 |
| 00140 Steroid hormone biosynthesis | 3 | 4 | 0.027 |
| 00550 Peptidoglycan biosynthesis | 2 | 4 | 0.027 |
| 00232 Caffeine metabolism | 2 | 4 | 0.027 |
| 00965 Betalain biosynthesis | 2 | 4 | 0.027 |
| 00281 Geraniol degradation | 1 | 3 | 0.020 |
| 00523 Polyketide sugar unit biosynthesis | 1 | 3 | 0.020 |
| 00363 Bisphenol degradation | 1 | 3 | 0.020 |
| 00624 Polycyclic aromatic hydrocarbon degradation | 1 | 3 | 0.020 |
| 00471 D-Glutamine and D-glutamate metabolism | 1 | 2 | 0.013 |
| 04977 Vitamin digestion and absorption | 1 | 2 | 0.013 |
| 01503 Cationic antimicrobial peptide CAMP resistance | 1 | 1 | 0.007 |
| 01053 Biosynthesis of siderophore group nonribosomal peptides | 1 | 1 | 0.007 |
| 00944 Flavone and flavonol biosynthesis | 1 | 1 | 0.007 |
| 00943 Isoflavonoid biosynthesis | 1 | 1 | 0.007 |
| 00254 Aflatoxin biosynthesis | 1 | 1 | 0.007 |
